# Relationship Between Sexual Behaviors with Non-committed Relationship Partners and COVID-19 Restrictions and Notification Rates: Results from a Longitudinal Study of Gay and Bisexual Men in Australia

**DOI:** 10.1007/s13178-022-00733-8

**Published:** 2022-05-28

**Authors:** Daniel Storer, Garrett Prestage, Hamish McManus, Lisa Maher, Benjamin R. Bavinton, Jeanne Ellard, Fengyi Jin, Steven Philpot, Martin Holt, Peter Saxton, Bridget Haire, Dean Murphy, Mohamed A. Hammoud

**Affiliations:** 1 The Kirby Institute, UNSW, Wallace Wurth Building, Sydney, NSW 2052 Australia; 2 Australian Research Centre in Sex, Health and Society, La Trobe University, Bundoora, Australia; 3 Centre for Social Research in Health, UNSW, Sydney, Australia; 4 Department of Social and Community Health, University of Auckland, Auckland, New Zealand

**Keywords:** Gay and bisexual men, COVID-19 restrictions, COVID-19 notifications, Sexual behavior, Lockdown

## Abstract

**Introduction:**

COVID-19 related lockdowns have impacted the sexual activity of gay and bisexual men (GBM). We investigated trends in sexual behaviors and the COVID-19 context in which they occurred (COVID-notification rates and jurisdictional restrictions) to understand changes in the duration and severity of periods of lockdown on the sexual behavior of Australian GBM.

**Methods:**

In an online, prospective observational study of 831 GBM from May 2020 to May 2021, we investigated associations between changes in sexual behavior among Australian GBM, lockdowns, and COVID-19 notification rates through weekly surveys from May 2020 to May 2021.

**Results:**

The mean age was 45.71 years (SD: 13.93). Most identified as gay (89.0%) and 10.2% were living with HIV. There was an overall increase in the mean weekly number of non-committed relationship partners (0.53–0.90, *p* < 0.001). The state of Victoria experienced a significant extended COVID-19 outbreak, accompanied by severe lockdown restrictions. In response, Victorian men’s partner numbers shifted three times, while elsewhere there was an overall gradually increasing trend.

**Conclusions:**

Less severe outbreaks with shorter lockdown periods, involving fewer and geographically contained, COVID-19 notifications were accompanied by non-significant changes in sex with non-relationship partners than more severe outbreaks over extended periods and larger geographical areas.

**Supplementary Information:**

The online version contains supplementary material available at 10.1007/s13178-022-00733-8.

## Introduction

Australiaʼs initial response to COVID-19 was characterized by restrictions on travel over the international border to all non-residents, with limits and restrictions on movement in and out of the country for residents. Similar to Taiwan and New Zealand that applied stringent international border restrictions soon after the pandemic began, this measure severely limited the possibility of the introduction of COVID-19 into Australia and resulted in a relatively low prevalence epidemic compared to other countries such as the UK and the USA. As of 2 May 2021, Australia reported a total of 29,826 cases compared to more than 4.2 million in the UK and almost 32.5 million in the USA (Public Health England, 2021; Australian Government Department of Health, 2021; Centers for Disease Control and Prevention, 2021). Domestically in response to the pandemic, Australiaʼs federal government is responsible for economic stimulus, procurement of COVID-19 vaccination supplies, and control of the international border. Australia’s six state and two territory governments have a mandate over hospitals and public health services and localized restrictions on movement, including control of domestic state and territory borders. It is Australian state and territory governments that have formulated and enforced jurisdictional public health policies throughout the pandemic in response to localized outbreaks and tailored these to the needs of their jurisdictions.

**Fig. 1:**
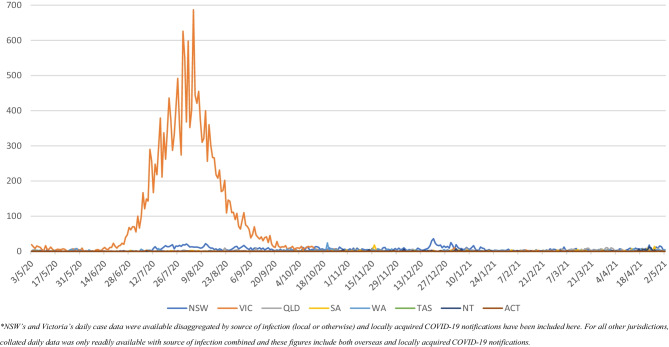
Daily new COVID-19 notifications in Australian states and territories (COVID-19 Data, 2021; Data.NSW, 2021; Department of Health and Human Services, 2021)*

However, states and territories have shouldered responsibilities of Australian residents returning from overseas and their quarantine requirements through jurisdictional hotel quarantine programs. It is through these hotel quarantine programs that Australia has experienced outbreaks to varying degrees throughout the country. Responses to these outbreaks are the responsibility of the state or territory governments and have resulted in varying degrees of restrictions on movement, including lockdowns (which are defined by ‘stay-at-home’ orders that narrowly mandate allowable reasons for leaving the household). A nationwide lockdown commenced 29 March 2020 (Prime Minister of Australia, 2020), and gradual easing of those restrictions began across jurisdictions in late April–early May 2020. At the time of writing, additional lockdowns across Australian jurisdictions have occurred in seven of the eight states and territories (New South Wales (NSW), Victoria, Queensland, Western Australia, South Australia, Australian Capital Territory, and the Northern Territory). People in Victoria’s capital city, Melbourne, experienced the longest and strictest lockdown in the country before 2 May 2021 (The Hon Daniel Andrews, Premier of Victoria, 2020a). A detailed description of these conditions has been described elsewhere (Pienaar et al., 2021). Importantly, COVID-19 infection rates varied across Australian states and territories, with differing patterns for timing, geographic extent, duration, and types of restrictions as a result (see Fig. 1).

**Fig. 2:**
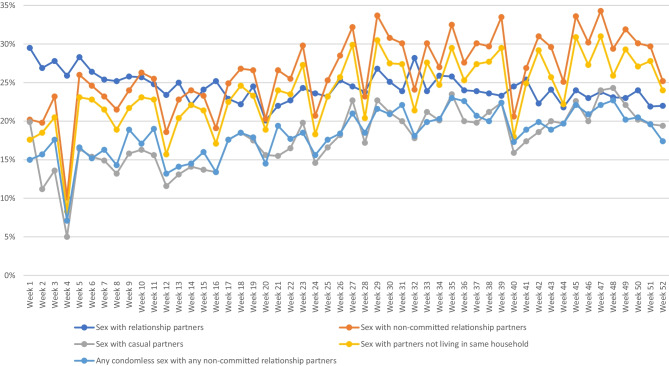
Proportions of men engaging in sex by partner type, sexual behavior in the previous 7 days for each study week

Under Australia’s state- and territory-based physical distancing restrictions introduced in some form from the end of March 2020, sexual contact outside the immediate household was initially prohibited until early April 2020 (COVID-19 National Incident Room Surveillance Team, 2020). Authorities in Australia’s two most populous jurisdictions, NSW and Victoria, clarified in April 2020 that visiting or meeting with an intimate partner was acceptable under the lockdown’s ‘stay-at-home’ orders (Purtill, 2020). Intense physical distancing measures in the form of lockdowns in response to more severe COVID-19 outbreaks, particularly periods of longer than a week, interrupt (physical) sexual partnering, including among gay and bisexual men (GBM) (Camargo et al., 2021; Hyndman et al., 2021; Reyniers et al., 2020; Sanchez et al., 2020; van Bilsen et al., 2021). Previous Australian research has demonstrated a substantial reduction in sexual partners within this population as a result of a national lockdown (Chow et al., 2020; Hammoud et al., 2020). However, it has been reported that GBM in some contexts have increased or not changed their sexual behavior with non-committed relationship partners (that is, ‘fuckbuddies’/friends with benefits and casual partners) during periods of lockdown or physical distancing restrictions (de Sousa et al., 2020; Starks et al., 2020; Stephenson et al., 2020). While evidence has mostly been restricted to specific time points in the pandemic (Linnemayr et al., 2020; Quinn et al., 2020; van Bilsen et al., 2021), it is plausible that as periods of lockdown change, in both time and severity of their restrictions in response to the perceived needs of the outbreak, then impacts on GBM’s sexual behavior will reflect these changes. To date, minimal research has reported longitudinal data collected at different time points during the pandemic but has demonstrated transient changes in sexual behavior among GBM (Jongen et al., 2021).

To understand changes in the duration and severity of periods of lockdown on the sexual behavior of Australian GBM, we investigated trends in sexual behaviors and the COVID-19 context in which they occurred (COVID-notification rates and jurisdictional restrictions).

## Methods

### Study Design and Procedures

Launched in 2014, the Flux Study is a national online, prospective observational study on GBM’s health. A detailed description of the study and its protocols has been published elsewhere (Hammoud et al., 2017). The study initially focused on use of illicit drugs, moving to attitudes and use of HIV pre-exposure prophylaxis (PrEP), and in 2020 it progressed to capture the experiences of the cohort in response to the COVID-19 pandemic. Part of this progression involved men responding to weekly surveys instead of surveys every 6 months. These surveys were sent every Sunday to capture their experience of COVID-19 from the preceding week in an ongoing basis in what were called weekly diaries beginning in May 2020. Information collected through a brief 5–10-min questionnaire captured essential information about sexual behavior, social connectedness, and access to healthcare. Every fourth week, participants responded to an extended 10–15-min survey to capture more detailed and contextual information including less common sex practices, such as group sex and sexualized drug use, sexual health checks, and practices employed to reduce transmission of COVID-19. Changes in behaviour from participants who responded to surveys prior to onset of the pandemic and the baseline of this stage of the study progression have been reported elsewhere (Hammoud et al., 2020).

Men were eligible if they were at least 16 years old, identified as gay or bisexual, or had sex with a man in the previous 12 months, and lived in Australia. Study promotion occurred online by advertising on a popular social media platform, Facebook, and LGBT and HIV community organizations across Australia, including recruitment of new participants in study weeks 8 (22–28 June 2020) and 37 (11–17 January 2021). Each week, participants who completed their weekly survey were entered into a raffle with three prizes: one $100 gift card and two $50 gift cards. Participants provided online informed consent, and enrolment was verified once participants activated a link via email. Ethical approval was granted from the Human Research Ethics Committee of UNSW Sydney.

### Measures

All weekly surveys included questions about sexual behavior. This included the number of male partners in the previous 7 days, whether they lived in the same household with those partners, whether those partners included a main regular male partner (boyfriend or husband), or any regular fuckbuddies or “friends with benefits.ˮ All other partners were considered casual partners for this analysis. We defined “non-committed relationship partnersˮ as fuckbuddies/ “friends with benefitsˮ and casual partners collectively, and “relationship partnersˮ as partners with whom participants had an ongoing committed relationship (boyfriend or husband) (Bavinton et al., 2016). Participants were asked to recall their sexual partners/behaviors aggregated over the previous week.

### Analysis

Joinpoint regression using the grid search method and with *p* values computed using the permutation procedure based on Monte Carlo methods for model selection was used to identify changes in trends of mean numbers of all non-relationship partners (Kim et al., 2000; Lerman, 1980). Software used was Joinpoint Regression Program Version 4.8.0.1. (Statistical Methodology and Applications Branch, 2021).

We reported periods of lockdown for Australian jurisdictions and jurisdictional COVID-19 notification rates and described how these compare with changes in trends identified in the joinpoint model. Periods of lockdown were defined by the implementation of “stay-at-homeˮ orders, where leaving the home was allowed for only four reasons: to seek or provide medical or compassionate care, for essential shopping, for work or study where this is not possible from home, and for exercise (Prime Minister of Australia, 2020).

We used relatively narrow time intervals (weekly) for joinpoint analyses in this study given the relatively high response rate per unit time (week), where even smaller jurisdiction categories used in the analysis averaged over 50 responses per week. This potentially increases the capacity of joinpoint selection to reflect multiple changes in trend by jurisdiction, especially for Victoria following changes in lockdown status of that jurisdiction over the analysis duration. However, this also has the potential to affect model selection and fit, as well as to lead to spuriously correlated trends if models are overspecified. Methodological steps taken to limit potential overspecification of models included restricted maximum number of joinpoints per model (4), as well as the minimum number of observations between consecutive joinpoints (3) or between joinpoints and study start and finish dates (3). Generally, broad (zero joinpoint) trends were observed in jurisdiction categories for fitted models suggesting limited potential spurious correlation. For comparison, we also used broader time intervals (monthly) for joinpoint analysis. We have chosen to present data on a weekly basis because of increased fidelity to observed changes across all jurisdictions over the study period.

We also conducted a random effects Poisson regression, univariate time predictor variable (weekly) fit model for each jurisdiction for interpretability and comparability with the joinpoint output. The model was guided by the joinpoint program to indicate inflexion point locations. The model for Victoria was fit piecewise, where a separate model was used for each segment between joinpoints. The rate ratios were annualized and presented as percentage changes to ensure format comparability with the joinpoint output.

### Sensitivity Analysis

A sensitivity analysis was conducted for the joinpoint regression model to control for age and level of retention in the cohort (completed at least 50% of weekly surveys). Because differences in study follow-up associated with age might potentially correlate with the primary endpoint, we conducted sensitivity analyses to look at outcomes by jurisdiction by dichotomized age group (< 35/ >  = 35), as well as limited to those participants who had completed at least 50% of survey responses.

Sensitivity analysis using monthly time intervals were qualitatively similar to the weekly models. Random effects Poisson regressions outputs were also qualitatively similar to outputs presented in the primary analysis.

Results.

### Sample Characteristics

A total of 831 GBM completed at least one survey between weeks 1 and 52 (3 May 2020–2 May 2021). Completed responses to each weekly survey over the 52 weeks ranged from 326 to 556 (see Supplementary Material Table 1). On average, each participant completed the survey in 28.5 of 52 weeks (SD = 20.32). Participants had a mean age of 45.71 years (SD: 13.93), ranging from 19 to 82 years (Table 1). Most men (89.0%) identified as gay with small proportions identifying as bisexual (5.9%) or another sexuality (2.4%) or gave no response (2.6%). About one in ten men reported being HIV-positive.

**Table 1:** Characteristics of sample (*N* = 831; includes all men who responded during any of weeks 1–52)

**Age in years**	
Median	45.00
Mean	45.71
SD	13.93
**Country of birth**	
Australia	623 (75.0)
New Zealand	26 (3.1)
Oceania	1 (0.1)
Asia	39 (4.7)
North America	22 (2.6)
South and Central American Countries	7 (0.8)
Europe	70 (8.4)
Middle East	2 (0.2)
Africa	6 (0.7)
No response	35 (4.3)
**Sexuality**	
Gay/homosexual	740 (89.0)
Bisexual	49 (5.9)
Other	20 (2.4)
No response	22 (2.6)
**Education**	
Less than year university	207 (24.9)
Undergraduate	296 (35.6)
Postgraduate	318 (38.3)
No response	10 (1.2)
**State of residence**	
NSW	370 (44.5)
Victoria	225 (27.1)
Queensland	101 (12.2)
Western Australia	39 (4.7)
South Australia	31 (3.7)
Tasmania	7 (0.8)
ACT	23 (2.8)
Northern Territory	11 (1.3)
Elsewhere/unknown	24 (2.9)
**HIV status**	
HIV positive	85 (10.2)
HIV negative	703 (84.6)
Unknown HIV status	43 (5.2)
Sexual behavior over 52 weeks*	
No sex in any week	168 (20.2)
Sex with relationship partner only	105 (12.6)
**Any sex with non-committed relationship partners**	
Up to 10% of weeks	93 (11.2)
10.1–45% of weeks	209 (25.1)
45.1–55% of weeks	42 (5.0)
55.1–90% of weeks	114 (13.7)
90.1–99.9% of weeks	24 (2.9)
Every week	77 (9.3)
CLAI-NR over 52 weeks*	
No CLAI-NR in any week	470 (56.5)
**Any CLAI-NR with non-committed relationship partners**	
Up to 10% of weeks	107 (12.8)
10.1–45% of weeks	148 (17.8)
45.1–55% of weeks	23 (2.8)
55.1–90% of weeks	51 (6.1)
90.1–99.9% of weeks	6 (0.7)
Every week	27 (3.2)

*Calculations are based on weeks due for each participant

One in five men (*n* = 168, 20.2%) reported no sex with male partners during the entire 52-week study period. About two-thirds (*n* = 559, 67.2%) reported sex with non-committed relationship partners, with over a quarter doing so in at least half of the surveys to which they responded. Among participants who reported sex with non-committed relationship partners, just over half (*n* = 470, 56.5%) of men reported no condomless anal intercourse with non-committed relationship partners (CLAI-NR). About two-fifths reported some CLAI-NR (*n* = 362, 43.5%), with over 10% doing so in at least half of the surveys to which they responded.

### Overall Trends in Sexual Behavior

A mean of 49.0% men per week engaged in any sex with any type of male partner with minimal variation over the 52-week study period (Supplementary Material Table 1). The proportion of men having sex with committed relationship partners fell slightly over the study period from 29.5% (week 1) to 22.0% (week 52; *p* =  < 0.001). Although there was an overall increase in sex with non-committed relationship partners, this was not a consistent increase, differing from week to week and ranged from 10.1 (week 4) to 34.3% (week 47) (Fig. 2).

Sex with casual partners in the previous 7 days changed little over time from 19.9% in week 1 (3–10 May) to 19.4% in week 52 (26 April–2 May 2021). However, the lower proportions tended to be observed in the earlier weeks of the study, between weeks 2 (11.2%, 11–17 May 2020) and 16 (13.4%, 17–23 August). There was a gradual increase in sex with non-household partners (i.e., non-committed relationship partners that did not reside with participants) over the study period. Only about 3% of men each week reported living in the same household as non-committed relationship partners.

Prevalence of CLAI-NR was inconsistent in the first half of the study period, from its lowest at 7.1% in week 4 (25–31 May 2020) to a peak of 19.4% in week 21 (21–27 September 2020) but remained steady at about one in five in the second half of the study period.

The mean number of partners overall varied across the study period, ranging from its lowest at 0.82 in week 1 (3–10 May 2020) to a peak of 1.57 in week 39 (25–31 January 2021), with a more consistent increase occurring in the latter half of the study period. The mean number of non-committed relationship partners gradually increased over the study period from 0.53 (week 1) to 0.92 (week 52).

Group sex behavior was collected every 4 weeks and reported for the previous four weeks. The proportion of men reporting group sex was also inconsistent over the study period, peaking early in the study at 15.2% in week 4 (25–31 May 2020).

Among the 413 men who responded in week 52, 9.9% (*n* = 41) indicated they were avoiding all sexual contact to reduce their risk of COVID-19. These 41 men reported a mean of 0.02 non-committed relationship sex partners in that week, compared with 1.03 partners among the remaining respondents in week 52 (*p* = 0.002). Similar trends were found in earlier weeks.

### Jurisdictional Trends in Sexual Behaviors, Lockdowns, and COVID-19 Notifications

Between 3 May 2020 and 2 May 2021, most Australian jurisdictions recorded no COVID-19 notifications for extended periods. When COVID-19 outbreaks did occur, they typically involved one, not all jurisdictions, and peak notifications in each jurisdiction varied, mostly for periods lasting no more than a few days. Joinpoint regression using permutation tests for the models found an overall continuous increase in the mean number of non-committed relationship partners over time (0.53–0.90, *p* < 0.001). Overall, the trends in the mean number of non-committed relationship partners varied across Australian jurisdictions (see Supplementary Material Table 2).

### Victoria

**Fig. 3:**
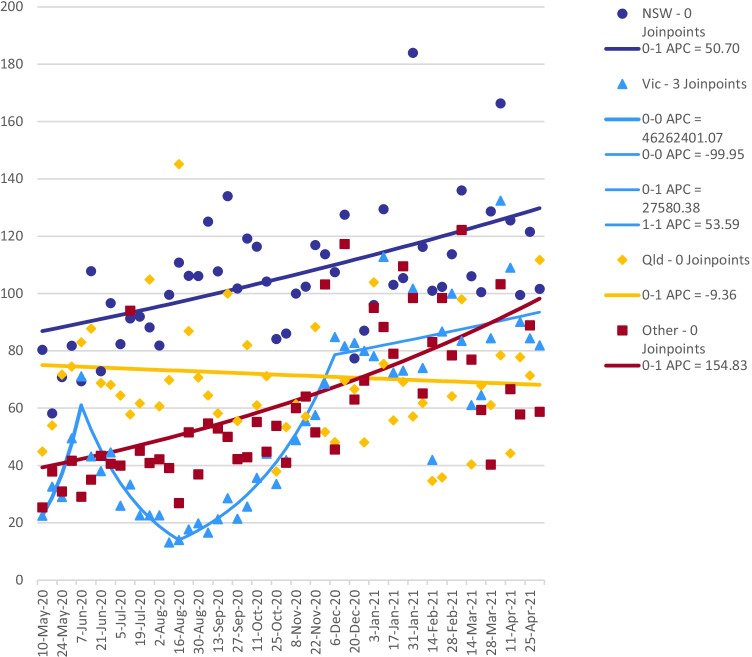
Multiple joinpoint models for all Australian jurisdictions and sex with non-romantic relationship partners

Over the study period, COVID-19 notifications in Victoria began to rise significantly from study week 8 (229 notifications, 22–28 June 2020) and peaked at 3125 during study week 13 (27 July–2 August 2020) (Fig. 1) which prompted a lockdown of metropolitan Melbourne and a neighboring local government area from study week 10 (6–12 July 2020) (Department of Health and Human Services, 2021) affecting approximately 83% of the state’s population (Australian Bureau of Statistics, 2021a, b; Mitchell Shire Council, 2021). This was followed by a state-wide lockdown, encompassing the remaining regional areas of the state, from study week 14 (3094 notifications, 3–9 August 2020). In week 19 (311 notifications, 7–13 September 2020), regional Victoria came out of lockdown, followed by metropolitan Melbourne in study week 22 (65 notifications, 28 September–4 October 2020) (Department of Health and Human Services, 2021). Although there were a number of weeks where metropolitan Melbourne was in lockdown and the rest of the state was not, the number of men outside of the lockdown during this time was too small to provide a reliable comparison of changes in behaviour. We observed three changes in the trend for the mean number of reported non-committed relationship partners from the joinpoint regression model. There was a trend increase from week 1 (85 notifications, 3–10 May 2020) to week 5 (15 notifications, 1–7 June 2020) (0.22–0.71, p 0.04), a trend decrease to week 16 (1,311 notifications, 17–23 August 2020) (0.71–0.18, *p* < 0.001), a trend increase to week 31 (0 notifications, 30 November–6 December 2020) (0.18–0.85, p 0.06), followed by a stable, slightly increasing, trend to week 52 (0 notifications, 26 April–2 May 2021) (0.85–0.82, p 0.61) (Fig. 3) (Department of Health and Human Services, 2021). A snap 5-day lockdown was also implemented for the entire state of Victoria across study weeks 41 (0.42, 13 notifications, 8–14 February 2021) and 42 (0.87, 5 notifications, 15–21 February 2021) (Department of Health and Human Services, 2021). The mean number of non-committed relationship partners remained much lower among Victorian participants compared to all other jurisdictions for the first half of the study period. However, mean partner number increased thereafter and peaked at 1.34 in week 48 (0 notifications, 18–24 January 2021) (see Supplementary Material Table 2) (Department of Health and Human Services, 2021) Fig. 4.

**Fig. 4:**
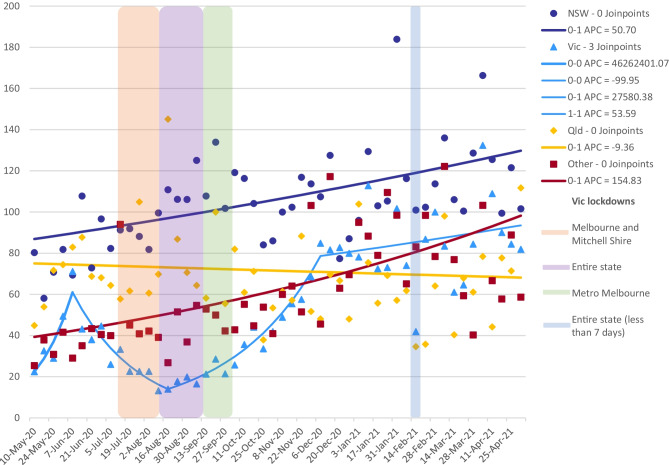
Multiple joinpoint models for all Australian jurisdictions, sex with non-committed relationship partners, and periods of lockdown for Victoria

**Fig. 5:**
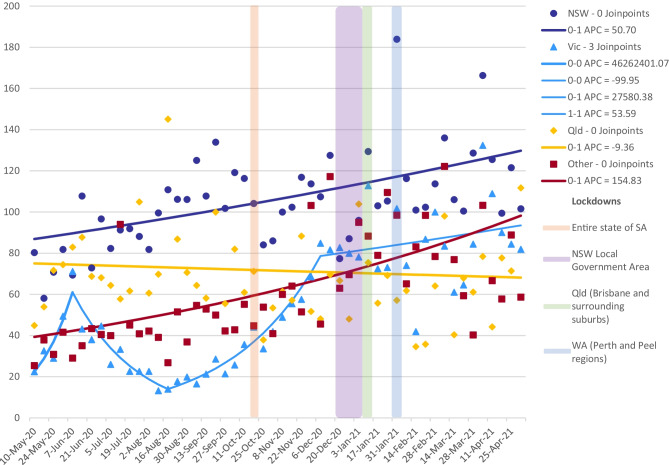
Multiple joinpoint models for all Australian jurisdictions, sex with non-committed relationship partners, and periods of lockdown for all jurisdictions (excluding Victoria)

### New South Wales

Over the study period, COVID-19 notifications in NSW varied but at no time reached similar levels to those observed in Victoria. Between study weeks 10 (16 notifications, 6–12 July 2020) to 21 (11 notifications, 14–20 September 2020), the state recorded a spike in notifications peaking at 92 notifications in week 13 (27 July–2 August 2020) (Fig. 1) (Data.NSW, 2021). No period of lockdown was introduced over this period. The mean number of non-committed relationship partners was at its lowest in week 13 (0.82, 92 notifications, 27 July–2 August) and peaked at 1.25 in week 18 (52 notifications, 31 August-6 September 2020) (see Supplementary Material Table 2) (Data.NSW, 2021). A localized lockdown of a specific local government area occurred from study weeks 33 (0.77, 88 notifications, 14–20 December 2020) to 36 (1.30, 19 notifications, 4–10 January 2021) (Data.NSW, 2021). A very small number of participants were living within the localized lockdown area, and, therefore, comparisons of changes in sexual behavior between those within the lockdown and outside were not reliable. We observed a continuously increasing trend over the study period in the joinpoint regression model for men in NSW (0.80–0.98, *p* < 0.001) (Fig. 5).

### Queensland

A snap 3-day lockdown occurred across study weeks 36 (0.77, 1 notification, 4–10 January 2021) and 37 (0.56, 1 notification, 11–17 January 2021) for the state’s capital, Brisbane, and surrounding local government areas due to cases in the community with the Alpha variant of the virus (Fig. 1) (Queensland Health, 2021a, b). The joinpoint regression model found little change in the mean number of reported non-committed relationship partners over the study period (0.45–1.12, p 0.50) (Fig. 5).

### Remaining Jurisdictions (Western Australia, South Australia, Tasmania, Australian Capital Territory, and Northern Territory)

Two lockdowns occurred in two of the remaining jurisdictions grouped for this analysis. The mean number of non-committed relationship partners represented here are across all five jurisdictions (see Supplementary Material Table 2). The state of South Australia had a snap lockdown in study week 29 (0.43, 24 notifications, 16–22 November 2020) that lasted for 4 days (SA Health, 2020a, b, c, d, e). Western Australia’s capital, Perth, and the neighboring Peel region entered a 5-day lockdown across study weeks 39 (0.98, 1 notification, 25–31 January 2021) and 40 (0.66, 0 notifications, 1–7 February 2021) due to a case in the community with the Alpha variant (Department of Health, 2021). Mean number of non-committed relationship partners for these remaining jurisdictions remained fairly stable over the study period, although there were large fluctuations week on week. For these jurisdictions, we observed a gradual increase over the study period in the joinpoint regression model (0.25–0.60, p 0.00) (Fig. 5).

### Sensitivity Analysis

In analyses restricted to younger men (those aged under 30 years) and analyses restricted to men who completed at least 50% of weekly surveys, these jurisdictional trends remained unchanged.

## Discussion

Differences in the severity and extent of localized outbreaks across jurisdictions appear to have affected trends in sexual behavior in ways that mirror those outbreaks. Shifts in Victorian GBM’s sexual behavior throughout COVID-19 restrictions were associated with local jurisdictional changes in COVID-19 notifications and the state’s lockdowns due to the more substantial outbreak that occurred in that state. Elsewhere, local outbreaks were more limited in daily case numbers, duration of lockdowns, and geographic impact there were gradual, mostly consistent, increases in sexual behavior over time. There was a strong association between believing that avoiding sexual contacts reduces the risk of COVID-19 infection and reported number of non-committed relationship sex partners. It is likely, therefore, that changes in sexual behavior reflected how imminent participants perceived the risk of infection to be.

During the initial weeks of the arrival of the pandemic in Australia, shortly after the implementation of Australia’s initial, national lockdown, there was an initial reduction in sexual behavior, particularly with non-committed relationship partners, among GBM (Hammoud et al., 2020). As weekly data collection began in early May 2020, when most Australian jurisdictions had commenced easing restrictions, case numbers remained very low across the country, until June 2020 when they began to increase in Victoria. Thus, the overall and jurisdictional trends we found during our data collection period were relative to the initial reductions observed before weekly survey data collection.

Trends in sexual behavior among Victorian men in this sample of Australian GBM tended to coincide with jurisdictional COVID-19 notifications and often slightly anticipated the longer jurisdictional or local lockdowns. Research conducted by Chow et al. (2021) also observed reductions in sex with casual partners and meeting casual partners face-to-face within a sample of men in Melbourne between the nationwide lockdown and the second lockdown of metropolitan Melbourne. In Victoria, however, localized lockdowns began relatively soon after the initial national lockdown, included substantial numbers of daily notifications, and, soon after, encompassed the entire state. Victorian men’s levels of sexual activity with non-relationship partners increased more gradually following the easing of restrictions and reduction in COVID-19 notifications, compared to the rapidity of their decrease when COVID-19 notifications were increasing, and lockdown restrictions were imposed as a response. COVID-19 outbreaks involving only small numbers of notifications over a few days, short-duration lockdowns, or those in restricted geographical areas appear to have a relatively limited impact on the overall trends in menʼs sexual behavior.

A localized lockdown occured in NSW, although based on far smaller notification numbers and restricted to one local government area for 20 days. We observed slight reductions in the mean number of non-committed relationship partners in NSW around the time of this lockdown (weeks 33–35, 0.77, 0.88, 0.96, 14 December 2020–3 January 2021). These reductions had little impact on the overall, gradually increasing trend in the mean number of non-committed relationship partners for NSW men. Given the small numbers of men who reported residing in a postcode of the locked down local government area, an analysis of sexual behaviors between men in and out of localized lockdowns during this period was not possible. However, the decrease in sex with non-committed relationship partners during this lockdown period provides evidence that a localized lockdown and outbreak can have a broader impact within the same jurisdiction among men subjected to the same overall public health policy environment. It should be noted that during this time, restrictions on visitors in the home were placed on Greater Sydney down to 10 from 24 December 2020 (The Premier, 2020a) and as low as five from 31 December 2020 (The Premier, 2020b) which would have also influenced the sexual behaviors of men outside the localized lockdown.

Notably, men in NSW experienced an easing of restrictions from the initial, national lockdown from study week 1 (3–10 May 2020) with the next form of lockdown in this state not occurring until 32 weeks later. This second period of lockdown only occurred within a relatively contained geographical area and timeframe and smaller notifications compared to the extended lockdown in Victoria. Furthermore, shorter snap lockdowns that occurred in Victoria, Queensland, Western Australia, and South Australia, while severe in the restrictions that were imposed, they were brought on by smaller COVID-19 notifications than the extended lockdown in Victoria and did not translate to changes in trends of sexual behavior.

These findings suggest that Australian GBM, particularly men in Victoria, were monitoring local COVID-19 notifications, adhering to physical distancing restrictions, and adjusting their sexual behavior with non-committed relationship partners in response to changes in the pandemic when they occurred. They also suggest that men were largely exercising caution when increasing sexual behavior with non-committed relationship partners as restrictions eased and notifications lowered. This was particularly evident following the extended lockdown in Victoria but was less apparent for shorter periods of lockdown in other states or even for the snap 5-day lockdown that occurred in ictoria during study weeks 41–42. The snap lockdowns in Queensland and Western Australia were not in response to increasing cases but following community transmission of the Alpha variant of SARS-CoV-2 out of hotel quarantine programs (Government of Western Australia, 2021; Queensland Government, 2021; Laschon, 2021).

Explicit “bubbleˮ arrangements for “people living alone and single parentsˮ for “social interactionˮ were introduced in Victoria as part of the initial stages of easing restrictions following the second, more restrictive lockdown (between 5 August and 14 September 2020) (The Hon Daniel Andrews, Premier of Victoria, 2020b, c). These arrangements permitted one nominated visitor to a single person or parent household for socializing (with the condition that face coverings be worn) (The Hon Daniel Andrews, Premier of Victoria, 2020a). These “bubbleˮ arrangements could have facilitated some sexual activity during this period.

To our knowledge, these are the only data describing GBM’s sexual behavior during the pandemic that has been collected weekly over 52 weeks. Our data are also the first to map changes in jurisdictional lockdowns and COVID-19 notifications and how they align with changes in sexual behavior with non-relationship partners among these men. It will be important to continue to monitor trends in CLAI-NR among GBM to also identify what, if any, prevention strategies they adopt over time and to understand their risk in relation to HIV and other sexually transmissible infections during the ongoing COVID-19 pandemic.

### Limitations

Data on biomedical HIV prevention and treatment were captured as part of weekly surveys among this cohort of men; however, these data go beyond the capacity of this analysis focused on sexual behavior over biomedical HIV prevention. The impacts of COVID-19 outbreaks and resulting restrictions to curb increases and the impacts on biomedical HIV prevention and treatment should be explored in future research. Although it could not have been anticipated, comparable weekly data on the sexual behavior of Australian GBM did not exist before the COVID-19 pandemic, including in previous iterations of the Flux Study. Also, weekly data collection commenced after the initial restrictions were implemented and not captured before May 2020. This study utilized an online convenience sample and findings may not be representative of all Australian GBM. The calculations in this analysis relied on recall of sexual behavior with partners in the previous 7 days, and we believe that this short timeframe reduced the impacts of recall bias. These data may also be subject to social desirability bias. Given restrictions and periods of lockdown discouraged people from meeting others outside of their household, particularly for non-committed relationship partners, it is plausible that men underreported sexual encounters, particularly outside the household and during times of lockdown. Participants were not asked each week about whether restrictions that were in place or number of local infections directly impacted their sexual behavior or choice of sexual partner. Therefore, direct relationships between restrictions or local notifications on sexual behavior or sexual partner choice are not possible. As mentioned earlier, this is a relatively older cohort compared to other Australian community-based samples of GBM and a larger sample of younger men may not have exhibited the same trends in sex with non-committed relationship partners (Bavinton et al., 2020; Holt et al., 2019).

## Conclusion

Sex with non-committed relationship partners among Australian GBM tended to increase overall since the initial restrictions on movement to curb the COVID-19 pandemic. Throughout increases in COVID-19 notifications and the implantation of severe restrictions, men in the state of Victoria responded by adjusting their sexual activity with non-committed relationship partners. Victorian men were generally slower to increase sexual activity as notifications decreased and restrictions eased, signaling a cautiousness among affected men. Outbreaks of COVID-19 involving less substantial notification numbers, more geographically contained and shorter periods of lockdown, had a less substantial impact on trends in sex with non-committed relationship partners.

## Supplementary Information

Below is the link to the electronic supplementary material.

Supplementary file1 (DOCX 35 KB)
