# Supplementary material for: Relationship Between Sexual Behaviors with Non-committed Relationship Partners and COVID-19 Restrictions and Notification Rates: Results from a Longitudinal Study of Gay and Bisexual Men in Australia
Source: Sex Res Social Policy. 2022 May 28;20(2):825–36. doi: 10.1007/s13178-022-00733-8 (PMC9142826; doi:10.1007/s13178-022-00733-8)
Supplement: Supplementary file 1 — Supplementary file1 (DOCX 35 KB) [file 13178_2022_733_MOESM1_ESM.docx]

*Table 1. Proportions of men engaging in sex by partner type, sexual behavior, and mean number of partners for each study week.*

| Date | Week Number | Number of responses | Any sexual contact | Sex with relationship partners in previous 7 days | Sex with non-committed relationship partners in previous 7 days | Sex with casual partners in previous 7 days | Sex with partners not living in same household in previous 7 days | Any condomless sex with any non-committed relationship partners in previous 7 days | Group sex in previous 4 weeks | Mean partner number (week) | Mean non-committed relationship partner number (week) |
| --- | --- | --- | --- | --- | --- | --- | --- | --- | --- | --- | --- |
| 10-May-20 | 1 | 387 | 46.8% | 29.5% | 20.2% | 19.9% | 17.6% | 15.0% |  | 0.82 | 0.53 |
| 14-May-20 | 2 | 396 | 43.7% | 26.9% | 19.8% | 11.2% | 18.5% | 15.7% |  | 0.74 | 0.47 |
| 24-May-20 | 3 | 397 | 45.2% | 27.8% | 23.2% | 13.6% | 20.5% | 17.6% |  | 0.82 | 0.54 |
| 31-May-20 | 4 | 397 | 49.6% | 25.9% | 10.1% | 5.0% | 8.3% | 7.1% | 15.2% | 0.92 | 0.66 |
| 07-Jun-20 | 5 | 385 | 50.6% | 28.3% | 26.0% | 16.4% | 23.1% | 16.6% |  | 0.94 | 0.66 |
| 14-Jun-20 | 6 | 382 | 47.6% | 26.4% | 24.6% | 15.4% | 22.8% | 15.2% |  | 1.04 | 0.77 |
| 21-Jun-20 | 7 | 362 | 47.2% | 25.4% | 23.2% | 14.9% | 21.5% | 16.3% |  | 0.84 | 0.59 |
| 28-Jun-20 | 8 | 509 | 50.8% | 25.2% | 21.5% | 13.2% | 18.9% | 14.3% | 8.4% | 0.96 | 0.71 |
| 05-Jul-20 | 9 | 488 | 46.5% | 25.8% | 24.0% | 15.8% | 21.7% | 18.9% |  | 0.86 | 0.60 |
| 12-Jul-20 | 10 | 510 | 49.0% | 25.7% | 26.3% | 16.3% | 23.1% | 17.1% |  | 0.97 | 0.72 |
| 19-Jul-20 | 11 | 499 | 46.3% | 24.8% | 25.5% | 15.6% | 22.8% | 19.0% |  | 0.89 | 0.63 |
| 26-Jul-20 | 12 | 516 | 46.3% | 23.4% | 18.6% | 11.6% | 15.7% | 13.2% | 7.0% | 0.88 | 0.64 |
| 02-Aug-20 | 13 | 505 | 44.0% | 25.0% | 22.8% | 13.1% | 20.4% | 14.1% |  | 0.81 | 0.56 |
| 09-Aug-20 | 14 | 525 | 43.7% | 22.1% | 24.0% | 14.1% | 22.1% | 14.5% |  | 0.86 | 0.63 |
| 16-Aug-20 | 15 | 512 | 44.3% | 24.1% | 23.3% | 13.7% | 21.4% | 16.0% |  | 0.99 | 0.75 |
| 23-Aug-20 | 16 | 493 | 48.8% | 25.2% | 19.1% | 13.4% | 17.1% | 13.4% | 7.1% | 0.96 | 0.70 |
| 30-Aug-20 | 17 | 506 | 45.4% | 22.9% | 24.9% | 17.6% | 22.5% | 17.6% |  | 0.90 | 0.67 |
| 06-Sep-20 | 18 | 504 | 45.8% | 22.2% | 26.8% | 18.5% | 24.6% | 18.5% |  | 1.00 | 0.78 |
| 13-Sep-20 | 19 | 503 | 46.3% | 24.5% | 26.6% | 17.5% | 23.3% | 17.9% |  | 0.94 | 0.70 |
| 20-Sep-20 | 20 | 512 | 44.9% | 20.1% | 20.3% | 15.6% | 18.9% | 14.5% | 7.0% | 1.07 | 0.87 |
| 27-Sep-20 | 21 | 504 | 45.6% | 22.0% | 26.6% | 15.5% | 24.0% | 19.4% |  | 0.86 | 0.64 |
| 04-Oct-20 | 22 | 496 | 45.4% | 22.7% | 25.5% | 16.5% | 23.5% | 17.7% |  | 1.01 | 0.77 |
| 11-Oct-20 | 23 | 492 | 51.2% | 24.3% | 29.8% | 19.8% | 27.3% | 18.5% |  | 1.02 | 0.78 |
| 18-Oct-20 | 24 | 478 | 48.5% | 23.6% | 20.7% | 14.6% | 18.3% | 15.6% | 7.2% | 0.98 | 0.74 |
| 25-Oct-20 | 25 | 482 | 46.5% | 23.2% | 25.3% | 16.6% | 23.2% | 17.6% |  | 0.85 | 0.61 |
| 01-Nov-20 | 26 | 478 | 50.8% | 25.3% | 28.5% | 18.2% | 25.7% | 18.4% |  | 0.90 | 0.65 |
| 08-Nov-20 | 27 | 482 | 52.4% | 24.5% | 32.2% | 22.7% | 29.9% | 21.0% |  | 1.01 | 0.77 |
| 15-Nov-20 | 28 | 466 | 52.8% | 23.8% | 23.2% | 17.2% | 20.4% | 18.5% | 8.6% | 1.04 | 0.80 |
| 22-Nov-20 | 29 | 463 | 54.9% | 26.8% | 33.7% | 22.7% | 30.5% | 21.6% |  | 1.15 | 0.89 |
| 29-Nov-20 | 30 | 455 | 49.5% | 25.1% | 30.8% | 21.1% | 27.5% | 20.9% |  | 1.18 | 0.93 |
| 06-Dec-20 | 31 | 435 | 51.5% | 23.9% | 30.1% | 20.0% | 27.4% | 22.1% |  | 1.11 | 0.87 |
| 13-Dec-20 | 32 | 556 | 57.4% | 28.2% | 24.1% | 17.8% | 21.4% | 18.1% | 11.5% | 1.38 | 1.09 |
| 20-Dec-20 | 33 | 326 | 51.2% | 23.9% | 30.1% | 21.2% | 27.6% | 19.9% |  | 1.03 | 0.79 |
| 27-Dec-20 | 34 | 433 | 49.4% | 25.9% | 27.0% | 20.1% | 24.7% | 20.3% |  | 1.04 | 0.78 |
| 03-Jan-21 | 35 | 435 | 51.8% | 25.8% | 32.5% | 23.5% | 29.5% | 23.0% |  | 1.20 | 0.94 |
| 10-Jan-21 | 36 | 434 | 55.8% | 24.0% | 27.6% | 20.0% | 25.3% | 22.6% | 11.3% | 1.38 | 1.14 |
| 17-Jan-21 | 37 | 435 | 50.8% | 23.9% | 30.1% | 19.8% | 27.4% | 20.7% |  | 1.11 | 0.87 |
| 24-Jan-21 | 38 | 444 | 51.8% | 23.6% | 29.7% | 21.2% | 27.7% | 20.0% |  | 1.16 | 0.92 |
| 31-Jan-21 | 39 | 451 | 53.4% | 23.3% | 33.5% | 22.4% | 29.5% | 22.4% |  | 1.57 | 1.34 |
| 07-Feb-21 | 40 | 456 | 51.0% | 24.5% | 20.6% | 15.9% | 18.0% | 17.3% | 6.8% | 1.15 | 0.91 |
| 14-Feb-21 | 41 | 454 | 48.1% | 25.4% | 26.9% | 17.4% | 24.9% | 18.9% |  | 1.01 | 0.76 |
| 21-Feb-21 | 42 | 452 | 50.4% | 22.3% | 31.0% | 18.6% | 29.2% | 19.9% |  | 1.12 | 0.90 |
| 28-Feb-21 | 43 | 438 | 50.9% | 24.1% | 29.6% | 20.0% | 25.7% | 18.9 % |  | 1.25 | 1.00 |
| 07-Mar-21 | 44 | 442 | 51.4% | 21.8% | 25.1% | 19.7% | 22.2% | 19.7% | 11.8% | 1.40 | 1.18 |
| 14-Mar-21 | 45 | 434 | 53.9% | 24.0% | 33.6% | 22.6% | 30.9% | 22.1% |  | 1.18 | 0.94 |
| 21-Mar-21 | 46 | 440 | 50.0% | 23.0% | 30.2% | 20.0% | 27.3% | 20.9% |  | 1.07 | 0.84 |
| 28-Mar-21 | 47 | 429 | 53.1% | 23.8% | 34.3% | 24.0% | 31.0% | 22.1% |  | 1.21 | 0.97 |
| 4-Apr-21 | 48 | 428 | 57.5% | 23.1% | 29.4% | 24.3% | 25.9% | 22.7% | 14.2% | 1.61 | 1.38 |
| 11-Apr-21 | 49 | 426 | 51.6% | 23.0% | 31.9% | 22.1% | 29.3% | 20.2% |  | 1.27 | 1.04 |
| 18-Apr-21 | 50 | 425 | 50.4% | 24.0% | 30.1% | 20.2% | 27.1% | 20.5% |  | 1.13 | 0.89 |
| 25-Apr-21 | 51 | 424 | 50.7% | 21.9% | 29.7% | 19.6% | 27.8% | 19.6% |  | 1.24 | 1.02 |
| 2-May-21 | 52 | 413 | 51.3% | 22.0% | 25.2% | 19.4% | 24.0% | 17.4% | 8.9% | 1.14 | 0.92 |

*Table 2. Mean number of non-relationship partners by study week and jurisdiction.*

|  | NSW | Victoria | Queensland | Other jurisdictions | Total sample |
| --- | --- | --- | --- | --- | --- |
| Week 1 | 0.80 | 0.22 | 0.45 | 0.25 | 0.53 |
| Week 2 | 0.58 | 0.33 | 0.54 | 0.38 | 0.47 |
| Week 3 | 0.71 | 0.29 | 0.72 | 0.31 | 0.54 |
| Week 4 | 0.81 | 0.49 | 0.75 | 0.42 | 0.66 |
| Week 5 | 0.69 | 0.71 | 0.83 | 0.29 | 0.66 |
| Week 6 | 1.06 | 0.43 | 0.88 | 0.35 | 0.77 |
| Week 7 | 0.73 | 0.38 | 0.69 | 0.43 | 0.59 |
| Week 8 | 0.96 | 0.45 | 0.68 | 0.41 | 0.71 |
| Week 9 | 0.83 | 0.26 | 0.64 | 0.40 | 0.60 |
| Week 10 | 0.91 | 0.33 | 0.58 | 0.94 | 0.72 |
| Week 11 | 0.92 | 0.23 | 0.62 | 0.44 | 0.63 |
| Week 12 | 0.89 | 0.22 | 1.02 | 0.41 | 0.64 |
| Week 13 | 0.82 | 0.23 | 0.61 | 0.42 | 0.56 |
| Week 14 | 1.00 | 0.13 | 0.70 | 0.39 | 0.63 |
| Week 15 | 1.11 | 0.14 | 1.45 | 0.27 | 0.75 |
| Week 16 | 1.05 | 0.18 | 0.83 | 0.53 | 0.70 |
| Week 17 | 1.06 | 0.20 | 0.71 | 0.37 | 0.67 |
| Week 18 | 1.25 | 0.17 | 0.64 | 0.55 | 0.78 |
| Week 19 | 1.08 | 0.21 | 0.58 | 0.53 | 0.70 |
| Week 20 | 1.34 | 0.29 | 1.02 | 0.50 | 0.87 |
| Week 21 | 1.02 | 0.21 | 0.56 | 0.42 | 0.64 |
| Week 22 | 1.19 | 0.26 | 0.82 | 0.43 | 0.77 |
| Week 23 | 1.16 | 0.36 | 0.61 | 0.55 | 0.78 |
| Week 24 | 1.05 | 0.42 | 0.71 | 0.43 | 0.74 |
| Week 25 | 0.84 | 0.34 | 0.38 | 0.54 | 0.61 |
| Week 26 | 0.86 | 0.42 | 0.53 | 0.41 | 0.65 |
| Week 27 | 1.00 | 0.49 | 0.61 | 0.60 | 0.77 |
| Week 28 | 1.04 | 0.56 | 0.57 | 0.64 | 0.80 |
| Week 29 | 1.17 | 0.58 | 0.88 | 0.52 | 0.89 |
| Week 30 | 1.14 | 0.69 | 0.52 | 1.03 | 0.93 |
| Week 31 | 1.08 | 0.85 | 0.48 | 0.46 | 0.87 |
| Week 32 | 1.28 | 0.82 | 0.67 | 1.22 | 1.09 |
| Week 33 | 0.77 | 0.83 | 0.68 | 0.63 | 0.79 |
| Week 34 | 0.88 | 0.80 | 0.48 | 0.72 | 0.78 |
| Week 35 | 0.96 | 0.78 | 1.04 | 0.95 | 0.94 |
| Week 36 | 1.30 | 1.13 | 0.77 | 0.88 | 1.14 |
| Week 37 | 1.03 | 0.73 | 0.56 | 0.79 | 0.87 |
| Week 38 | 1.05 | 0.73 | 0.69 | 1.10 | 0.92 |
| Week 39 | 1.84 | 1.02 | 0.57 | 0.98 | 1.34 |
| Week 40 | 1.17 | 0.75 | 0.54 | 0.66 | 0.91 |
| Week 41 | 1.01 | 0.42 | 0.35 | 0.83 | 0.76 |
| Week 42 | 1.02 | 0.87 | 0.36 | 0.98 | 0.90 |
| Week 43 | 1.14 | 1.00 | 0.64 | 0.78 | 1.00 |
| Week 44 | 1.37 | 0.84 | 1.00 | 1.22 | 1.18 |
| Week 45 | 1.07 | 0.61 | 0.40 | 0.77 | 0.94 |
| Week 46 | 1.00 | 0.65 | 0.68 | 0.59 | 0.84 |
| Week 47 | 1.29 | 0.84 | 0.61 | 0.40 | 0.97 |
| Week 48 | 1.66 | 1.34 | 0.76 | 1.03 | 1.38 |
| Week 49 | 1.26 | 1.09 | 0.43 | 0.67 | 1.04 |
| Week 50 | 0.99 | 0.90 | 0.78 | 0.58 | 0.89 |
| Week 51 | 1.22 | 0.84 | 0.76 | 0.89 | 1.02 |
| Week 52 | 0.98 | 0.82 | 1.12 | 0.60 | 0.92 |
